# *Aspergillus ficuum* phytase activity is inhibited by cereal grain components

**DOI:** 10.1371/journal.pone.0176838

**Published:** 2017-05-04

**Authors:** Zelalem Eshetu Bekalu, Claus Krogh Madsen, Giuseppe Dionisio, Henrik Brinch-Pedersen

**Affiliations:** Department of Molecular Biology and Genetics, Research Center Flakkebjerg, Aarhus University, Slagelse, Denmark; Nanjing Agricultural University, CHINA

## Abstract

In the current study, we report for the first time that grain components of barley, rice, wheat and maize can inhibit the activity of *Aspergillus ficuum* phytase. The phytase inhibition is dose dependent and varies significantly between cereal species, between cultivars of barley and cultivars of wheat and between *Fusarium graminearum* infected and non-infected wheat grains. The highest endpoint level of phytase activity inhibition was 90%, observed with grain protein extracts (GPE) from *F*. *graminearum* infected wheat. Wheat GPE from grains infected with *F*. *graminearum* inhibits phytase activity significantly more than GPE from non-infected grains. For four barley cultivars studied, the IC_50_ value ranged from 0.978 ± 0.271 to 3.616 ± 0.087 mg×ml^-1^. For two non-infected wheat cultivars investigated, the IC_50_ values were varying from 2.478 ± 0.114 to 3.038 ± 0.097 mg×ml^-1^. The maize and rice cultivars tested gaveIC_50_ values on 0.983 ± 0.205 and 1.972 ± 0.019 mg×ml^-1^, respectively. After purifying the inhibitor from barley grains via Superdex G200, an approximately 30–35 kDa protein was identified. No clear trend for the mechanism of inhibition could be identified via Michaelis-Menten kinetics and Lineweaver-Burk plots. However, testing of the purified phytase inhibitor together with the *A*. *ficuum* phytase and the specific protease inhibitors pepstatin A, E64, EDTA and PMSF revealed that pepstatin A repealed the phytase inhibition. This indicates that the observed inhibition of *A*. *ficuum* phytase by cereal grain extracts is caused by protease activity of the aspartic proteinase type.

## Introduction

Phytases (myoinositol hexakisphosphate phosphohydrolase; EC 3.1.3.26 and EC 3.1.3.8) are phosphatases that initiate the sequential liberation of orthophosphate groups from phytate (myoinositol 1, 2,3,4,5, 6-hexakisphosphate). Phytate is the major storage form of phosphorous in plant seeds contributing up to 70% of the total phosphorus reserve [1] and 1–5% (dry w/w) of cereal grains, legume seeds, oilseeds, pollen and nuts [2]. In mature seeds, it exists as a mixed salt of K^+^, Ca^2+^, Mg^2+^ and Zn^2+^, called phytate/ phytin. In small grain cereals, about 90% of the phytate is located in the aleurone layer. The remaining ~10% is found in the scutellum [3]. Monogastric animals like pigs and poultry have basically no phytase activity in their digestive tract, and the phytase level of the mature plant seed is most often inadequate for efficient phytate hydrolysis in feed [4]. In consequence, most of the seed phytate in feed remains non-digested and is secreted and spread with the manure to the agricultural soils and eventually to the aquatic environment causing algal growth and eutrophication. Moreover, as chelator of nutritional important minerals, phytate is considered the major anti-nutritional factor for the bioavailability of micronutrient metals and contributes to mineral depletion and deficiencies in human populations that rely on whole grains and legume-based products as staple foods [5].

A series of strategies have been devised to improve the bioavailability of phosphate in animal feed and to reduce the environmental load. One of these is to add microbial phytase to feed and thereby enhance the release of phosphate from phytate. The commercial potential of this strategy has stimulated a large body of research and development activities to identify microbial phytases with favourable catalytic properties. Phytases from a range of different microorganisms such as *Escherichia coli* (i. e. Quantum, Quantum Blue and Phyzyme XP), *Buttiauxella* sp. (i. e. AxtraPHY), *Citrobacter braakii* (i.e. Ronozyme Hiphos), *Peniophora lycii* (i.e. Ronozyme NP) and *Aspergillus niger* (i. e. Nathuphos) have been commercialized. Among these, *A*. *niger* is also a known pathogen in cereals.

The filamentous ascomycete fungi *Aspergillus niger* is one of the most common species of the genus *Aspergilli* and cause the black mold diseases in fruits, vegetables and cereals [6]. It is mainly associated with postharvest decay in stored products and produces potential carcinogenic mycotoxins [7]. *A*. *niger* produces a wide array of hydrolytic and oxidative enzymes involved in the breakdown of host tissues [6], including phytase [8,9]. *A*. *ficuum* phytase is one of the most important industrial phytases. It has been thoroughly biochemically characterized [10] and its crystal structure has been published [11].

Several reports have described that the efficiency of microbial proteases and xylanases can be reduced significantly due to the presence of inhibitors in the feed crops [12,13]. Plants have evolved inhibitors of pathogenic microbial enzymes as defense components. Numerous inhibitors of microbial enzymes have been identified and characterized from plants [14–16]. *A*. *ficuum* phytase activity is known to be inhibited by cations such as Cu^2+^, Hg^2+^, Zn^2+^, Fe^2+^ and Fe^3+^ [17]. However, proteinaceous inhibitors of microbial phytases have so far never been reported in plants. Here, we describe for the first time the inhibition of *A*. *ficuum* phytase by cereal grain protein extracts. We also investigate variations in the inhibitory effect between cereals and cultivars, and the pathogen inducibility of phytase inhibitors and study the mechanism of phytase inhibition. The implication of a so far unknown phytase inhibitor, in varying levels, in food and feed and the possible potentials of a cereal inhibitor of pathogen phytase activity are discussed.

## Materials and methods

### Plant materials and reagents

Cultivars of winter wheat (*Triticum aestivum L*., cv. SJ8575204) and barley (*Hordeum vulgare* L., cv. ‘SJ111884’, ‘Matros’, ‘Invictus’ and ‘Agulatus’) were grown at Sejet Plant Breeding, Denmark. Commercial cultivars were included for maize (*Zea mays* cv. Delicata) and rice (*Oryza sativa* cv. Nipponbare). *F*. *graminearum* infected and non-infected grains of a wheat cultivar (*T*. *aestivum L*., cv. ‘Skalmeje’) were kindly provided by Lise Nistrup, Department of Agroecology, Aarhus University. Reagents including *A*. *ficuum* phytase (Sigma P-9792) and sodium phytate (from rice; Sigma P-8810) were supplied by Sigma.

### Preparation of grain extracts for inhibition studies

Grains were ground to a fine powder using a rotary mill (IKA^®^ Tube mill control). Grain cell-free proteins were extracted in 1:10 (w/v) 25mM sodium acetate buffer (pH 5.5) containing 0.1mM CaCl_2_, by constant shaking (300–350 rpm) at 25°C for 1h. The supernatant was collected by centrifugation (3392×g, for 30 minutes at 4°C) and used as grain protein extract (GPE) for the inhibition study.

### Enzyme inhibition assay

Phytase activity and its inhibition was measured according to ammonium-molybdate method [18]. In brief, 100 μl of GPE (0–2 mg ml^-1^) was incubated with 10 μl (2.5 U ml^-1^) of *A*. *ficuum* phytase, 1 mM sodium phytate and 400 μl of 25 mM sodium acetate buffer (pH 5.5) containing 0.1mM CaCl_2_, at 37°C for 1 hour. The reaction was terminated by adding 800 μl of stop solution (20mM ammonium heptamolybdate, 5mM ammonium vanadate and 6% nitric acid to the final concentration) to the reaction mixture. After centrifugation (4226×g, 5 min), the absorbance of the supernatant was measured at 415 nm using 96 well plate reader (Epoch, BioTek, USA). The residual phytase activity under different GPE concentrations was determined relative to a blank sample.

### IC_50_ value and kinetics of phytase inhibition

The 50% inhibitory concentration (IC_50_) was calculated from the dose–response curve obtained by plotting the percentage of phytase inhibition versus increasing concentrations of GPE (0–2 mg ml^-1^). Using the linear equation from the graphs, the IC_50_ values were determined taking the response (percentage of phytase inhibition) as 50%. The kinetic constants against *A*. *ficuum* phytase were determined by pre-incubating the enzyme in the presence of crude extract (1 mg ml^-1^) for 15 min at 25°C, followed by 45 min incubation at 37°C with increasing concentrations of sodium phytate substrate (0–2 mM). The activity of the enzyme was measured at four time points (0, 15, 30 and 45 min). The Michaelis-Menten constant (*Km*) and maximum velocity (*Vmax*) values were calculated by using Sigma Plot 11.0, Exploratory Enzyme Kinetics Module (Systat Software Inc., USA). The type of inhibition was determined from the Lineweaver-Burk plot. The kinetics of phytase inhibition was performed for the representatives of cereal species.

### Gel filtration chromatography of inhibitors from barley

Proteins were fractionated from the GPE of barley cv. Invictus using an ÄKTA fast protein liquid chromatography (FPLC) device equipped with a Superdex G200 (10/300 GL) column. The following protein standards were used for calibration of the column and estimating the apparent molecular weight of eluted fractions: 1) blue dextran (2000 kDa), 2) conalbumin (75 kDa), 3) ovalbumin (43 kDa), 4) carbonic anhydrase (29 kDa), 5) ribonuclease A (13.7 kDa) and 6) aprotinin (6.5 kDa). Protein standards were loaded and resolved separately into Superdex G 200 using isocratic elution in Buffer A (50 mM Na-acetate buffer pH 5.0 and 0.2 M NaCl). Briefly, proteins from GPE were precipitated using 60% ammonium sulphate at 4°C. The precipitate was re-suspended in 50 ml of 25 mM acetate buffer pH 4.5 and 1ml of resuspended pellet containing 20 mg ml^-1^ was loaded directly into the Superdex G 200. Proteins were eluted using an isocratic elution in Buffer A and ÄKTA FPLC specific Unicorn program. After 2 h of elution, each 2 ml fractions were collected and assayed for *A*. *ficuum* phytase inhibition.

### Treatment of FPLC fractions with protease inhibitors

The phytase inhibiting FPLC fractions were incubated with the following protease inhibitors at the indicated final concentration: 100 μM pepstatin A, 50 μM E-64, 5 μM EDTA (ethylenediaminetetraacetic acid) and 1 mM PMSF (phenylmethylsulfonyl fluoride). A mixture containing the 100 μl inhibitor fraction (1 mg ml^-1^), 10 μl *A*. *ficuum* phytase (2.5 Uml^-1^), 1mM sodium phytate and protease inhibitors, as stated above, were gently mixed and incubated in 400 μl of 25 mM acetate buffer pH 5.5 at 37°C for 1h. For blank samples, the inhibitors were substituted by an appropriate volume of 25 mM acetate buffer, pH 5.5. The effect of pre-incubation on the activity of the inhibitor was studied for pepstatin A. In the pre-incubation experiments, FPLC fractions were incubated in 100 μM pepstatin A at room temperature for 1 h. *A*. *ficuum* phytase (2.5 Uml^-1^) and 1mM sodium phytate substrate were added to the pre-incubated mix and further incubated for 1 h at 37°C. Control samples were prepared by incubating *A*. *ficuum* phytase (2.5 Uml^-1^) and 1 mM sodium phytate in sodium acetate buffer pH 5.5. Reactions were terminated with 800 μl stop solution and the absorbance was measured at 415 nm after centrifugation (4226×g, 5 min).

### Protein determination

The protein concentration was determined based on the method of [19] using Bovine serine albumin (BSA, Sigma) as a standard.

### Statistical analysis

All experiments were carried out in triplicates. Two-way analysis of variance (ANOVA) was used to compare the data with statistical significance considered as *P*< 0.05 (SigmaPlot11.0).

## Results

### Phytase inhibitory activity

**Fig 1:**
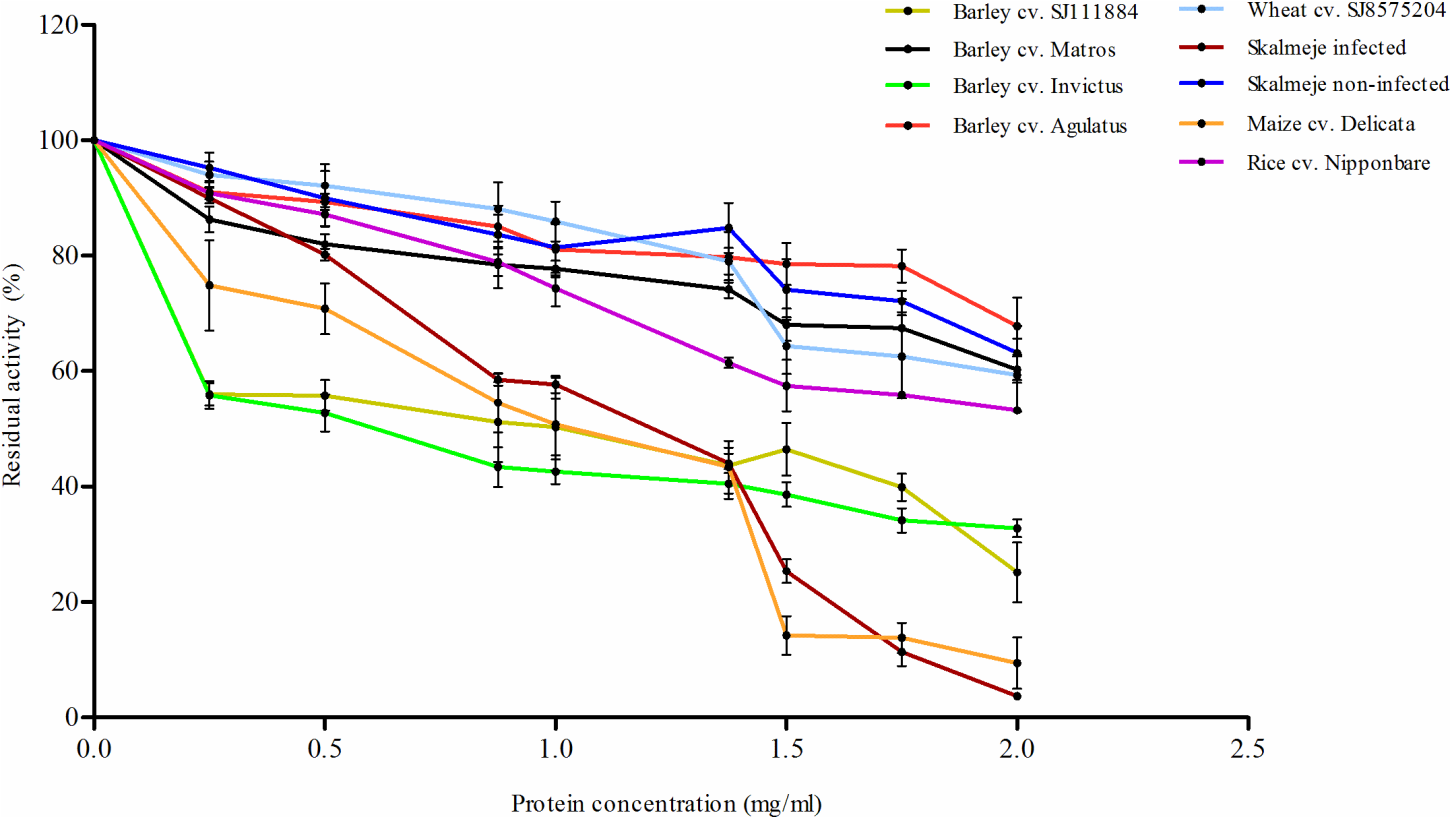
The effect of increasing concentrations of crude protein extracts of representatives of cereal species on the activity of *A*. *ficuum* phytase. Different cultivars of cereal species are represented with different coloured solid lines. Results are averages of three replicates and the differences among replicates are indicated with error bars. 100% residual activity is equivalent to 2.5 Uml^-1^ activity.

GPE´s from all grain samples caused a dose-dependent inhibitory effect on *A*. *ficuum* phytase activity (Fig 1). Moreover, there were different inhibition among species, barley and wheat cultivars and between *Fusarium* infected and non-infected wheat grains. The highest end point inhibitory effects were seen for maize, barley SJ111884 and Invictus cultivars and for *Fusarium* infected wheat. The barley cultivars Invictus and SJ111884 had clearly stronger inhibitory effects compared to the other two cultivars Agulatus and Matros. The inhibition by the two non-infected wheat cultivars was at the same level as the two less inhibiting barley cultivars Agulatus and Matros.

For wheat, pathogen-inducibility of phytase inhibitors was examined using GPE from *F*. *graminearum* infected and non-infected grains of cv. Skalmeje. The result showed that *Fusarium* infection has a clear positive inducing effect on the inhibition of A. *ficuum* phytase. More than 90% inhibition of phytase activity was obtained in the presence of 2 mg ml^-1^ proteins from *Fusarium* infected grains whereas GPE from the non-infected grains only reduced the phytase activity of about 35%.

### Kinetics of phytase inhibition

The IC_50_ values for *A*. *ficuum* phytase inhibition are shown in Table 1. The significantly lowest IC_50_ values were obtained from barley grains cv. Invictus, maize and *Fusarium* infected wheat cv. Skalmeje. The highest IC_50_ values were recorded for GPE of barley cv. Agulatus, followed by GPE from non-infected wheat cv. Skalmeje. All four barley cultivars tested had significantly different IC_50_ values. Moreover, also the two non-infected wheat cultivars had significant differences in the IC_50_ values. As described above, *Fusarium* infection significantly accelerated the inhibition of the *A*. *ficuum* phytase activity. Hence, to cause 50% inhibition, three times more concentrated extract of Fusarium non-infected grains was required compared to Fusarium infected grains.

**Table 1:** The 50% inhibitory concentration (IC_50_) of representatives of cereal species, barley cultivars and Fusarium infected wheat cultivar.

Seed extracts		Cultivars	IC_50_ (mg ml^-1^)
**Species**	Barley	cv. SJ111884	1.319 ± 0.298 ^b^
		cv. Matros	2.678 ± 0.058 ^d^
		cv. Invictus	0,978 ± 0,271 ^a^
		cv. Agulatus	3.616 ± 0.087 ^f^
	Wheat	cv. SJ8575204	2.478 ± 0.114 ^d^
		cv. Skalmeje (non-infected)	3.038 ± 0,097 ^e^
		cv. Skalmeje (Infected*)	1.072 ± 0.024 ^a^
	Maize	cv. Delicata	0.983 ± 0.205 ^a^
	Rice	cv. Nipponbore	1.972 ± 0.019 ^c^

*Grains of a wheat cultivar Skalmeje infected with *F*. *graminearum*. The letters indicate the level of significant differences (P>0.05).

**Fig 2:**
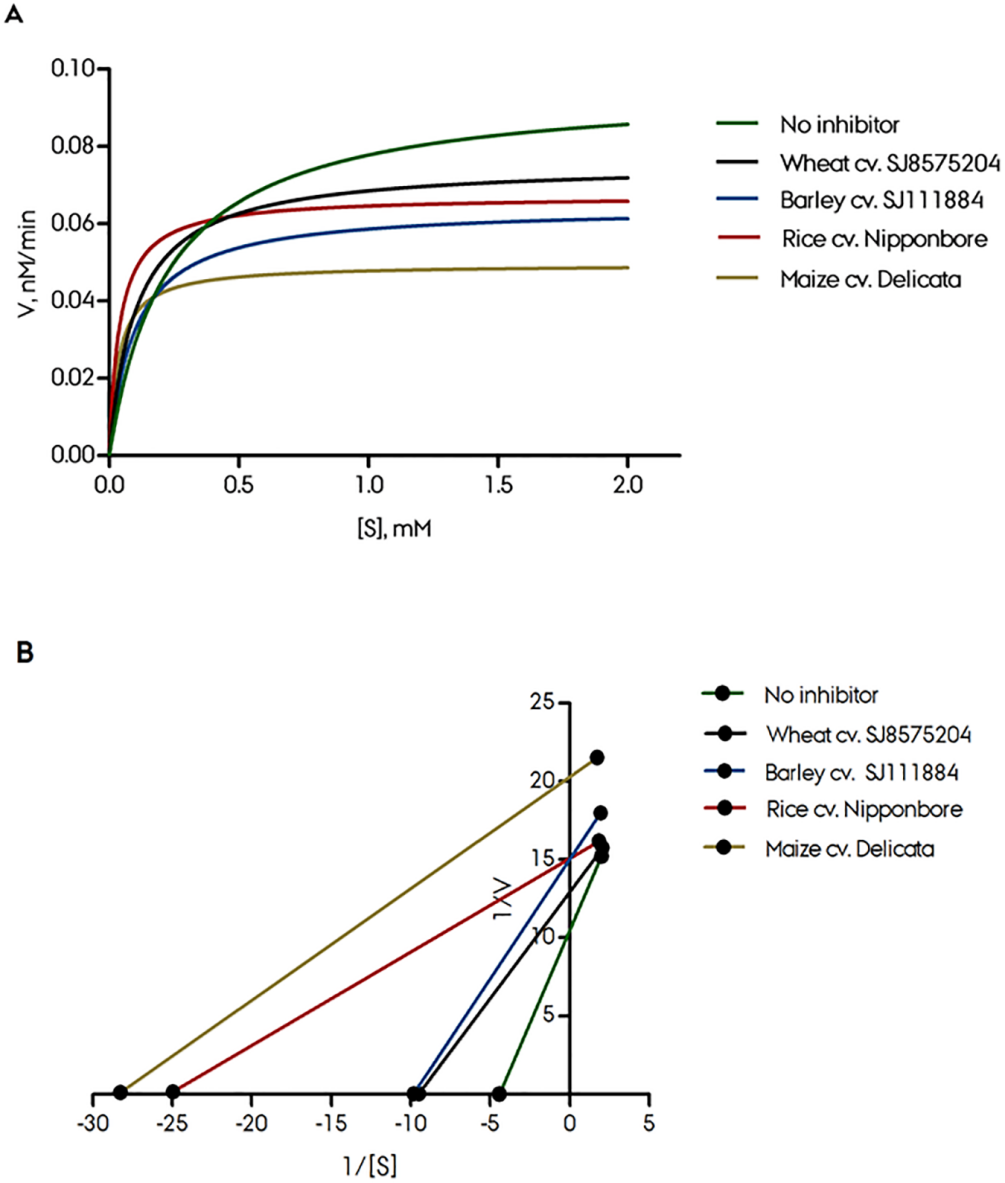
Mode of inhibition of *A*. *ficuum* phytase by crude protein extract of cereal species. (A) Michael-Menten plot and (B) Lineweaver-Burk plot.

In order to assess the type of phytase inhibition, enzyme kinetics was implemented by incubating a constant amount of *A*. *ficuum* phytase in the presence of GPE (1 mg ml^-1^) and increasing concentration of phytic acid substrate (0–2.0 mM). Enzyme kinetic parameters were determined from the Michaelis-Menten and Lineweaver-Burk plots (Fig 2A and 2B). The assessment was performed under the assumption that the significantly different inhibition levels of the samples are caused by different levels of the same inhibitor. The Michaelis-Menten plot produced *Km* and *Vmax* values respectively for no inhibitor control on 0.2267 mM and 0.0954 nM× min^-1^, for maize 0.03526 mM and 0.04945 nM× min^-1^, rice 0.04015 mM and 0.06714 nM× min^-1^, winter wheat (SJ8575204) 0.1028 mM and 0.07553 nM× min^-1^, and for barley (SJ111884) 0.09806 mM and 0.06434 nM× min^-1^. The highest velocity was observed in the absence of inhibitors or crude extracts. The type of inhibition of GPE against *A*. *ficuum* phytase was examined from the Lineweaver-Burk plot. As judged from K_m_ and V_max_ values and the Lineweaver-Burk plot (Fig 2B) it’s at this stage not clear if the inhibition is due to a general competitive, uncompetitive or noncompetitive inhibition mechanism. In detail all the samples except barley GPE showed a competitive inhibition. The trend could not be easily visible due to mixed type of competitive inhibition (regression lines will meet in the positive quadrant) (Fig 2B). Between barley and no-inhibitor the regression line seems to be almost parallel, hence the trend is towards uncompetitive inhibition (Fig 2B).

### FPLC fractionation of GPE

**Fig 3:**
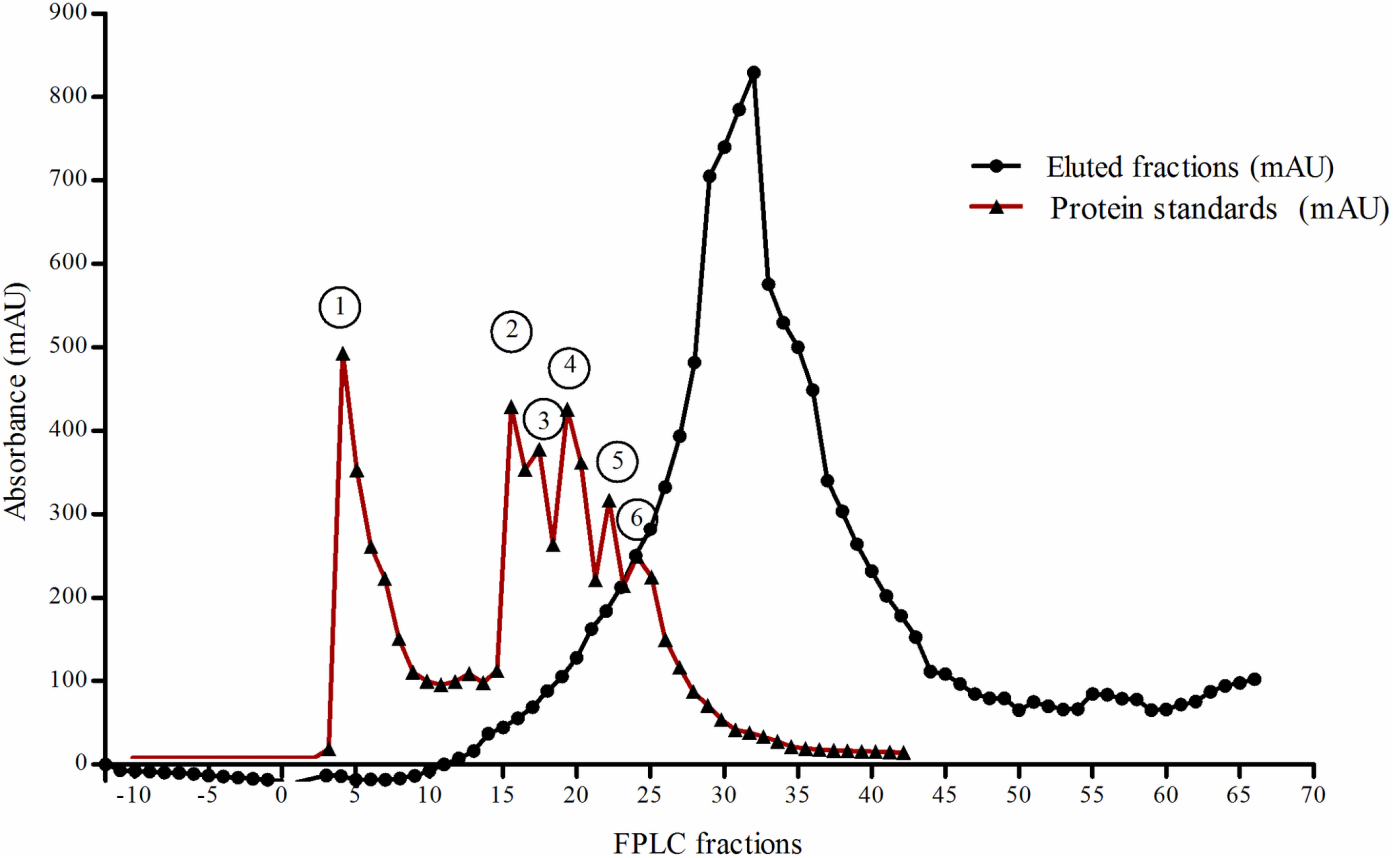
Estimation of the molecular weight of FPLC fractions eluted from Superdex G-200 using protein standards. The following protein standards were used: 1) blue dextran (2000 kDa), 2) conalbumin (75 kDa), 3) ovalbumin (43 kDa), 4) carbonic anhydrase (29 kDa), 5) ribonuclease A (13.7 kDa) and 6) aprotitin (6.5 kDa).

**Fig 4:**
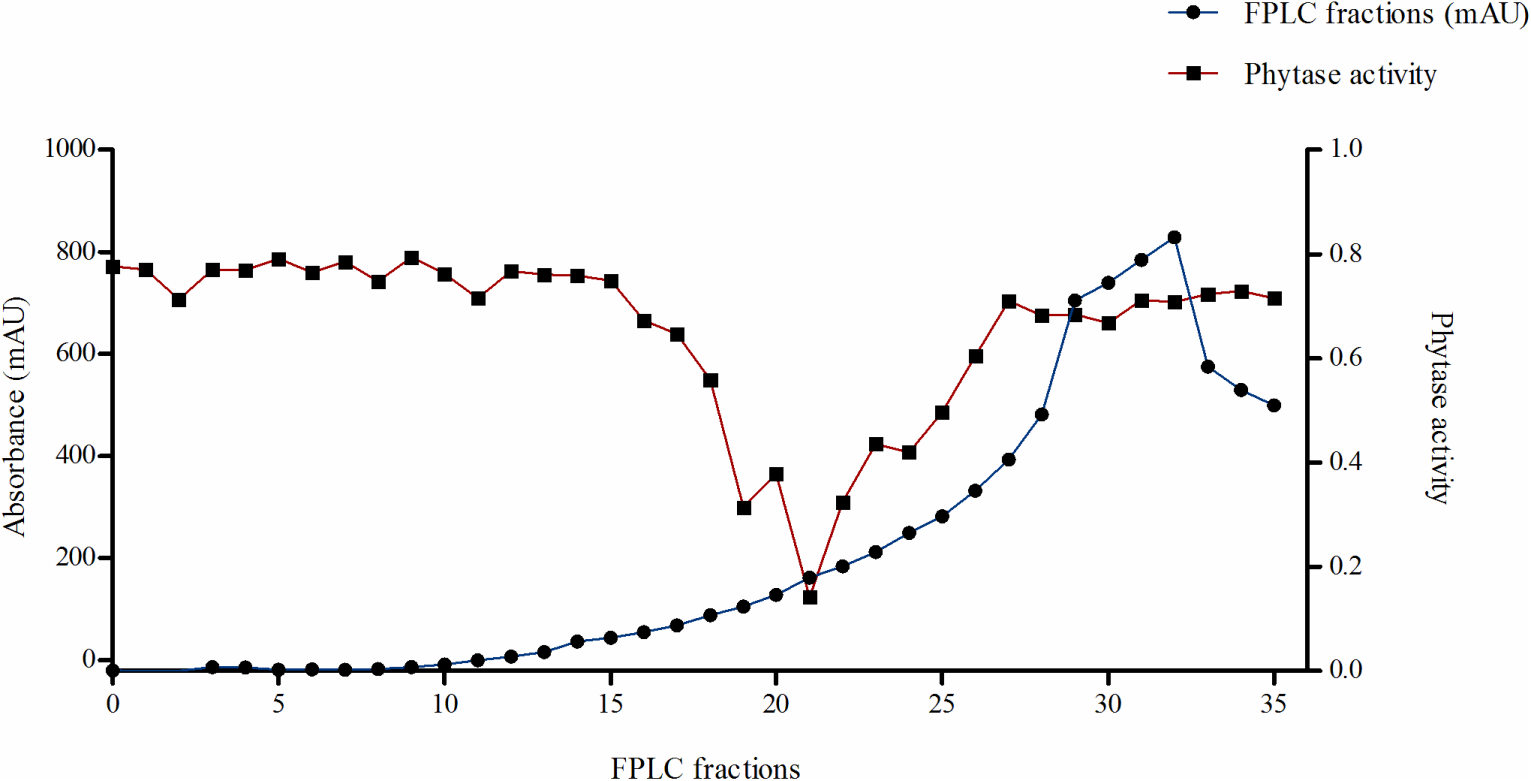
FPLC chromatogram of phytase inhibitory fractions (blue line) and the corresponding activity assay (red line) of fractions eluted from Superdex G-200.

Further identification of a proteinaceous phytase inhibitor was carried out by purifying from the GPE of barley cv. Invictus. Gel filtration of ammonium precipitated proteins was used to fractionate inhibitory components based on their apparent molecular weight. The gel filtration chromatogram for barley GPE was produced using a Superdex G 200 column. The eluted fractions formed a distinct elution peak with an estimated maximum at a molecular weight of about 6.5 kDa (Fig 3). In new inhibition studies focusing on fractions 14 to 35 we saw a significant inhibition of *A*. *ficuum* phytase ranging from 16.5 to 84.4% inhibition (Fig 4). The molecular weight of the peak inhibitory fraction (#21) was approximately 30–35 kDa.

### Protease or inhibitor treatments

**Fig 5:**
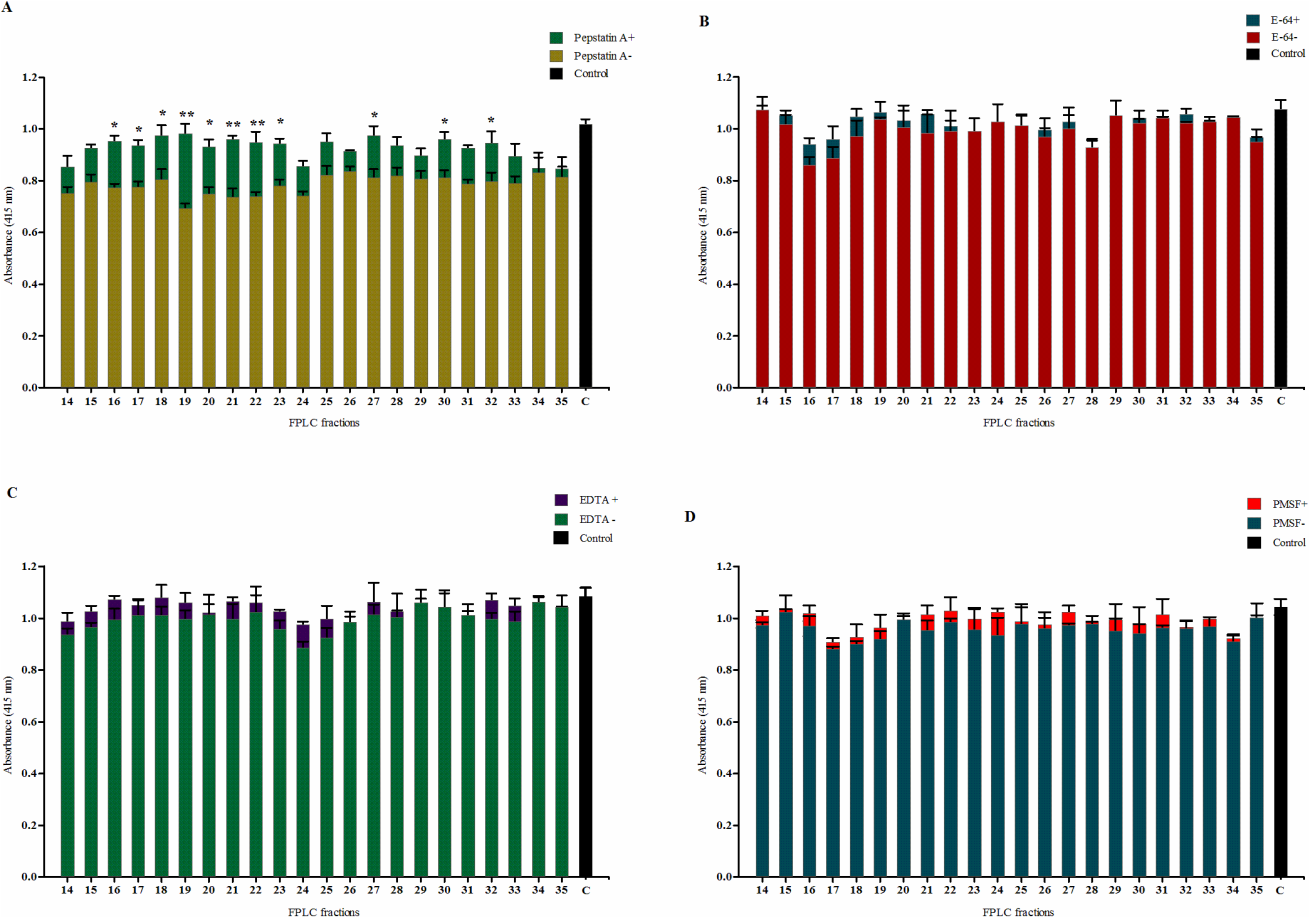
The effect of generic inhibitors of the main proteases on the activity of inhibitory FPLC fractions. Panels: A) Pepstatin A, B) E-64, C) EDTA, and D) PMSF. The bar “C” represents the blank sample, (–) and (+) represent the absence and presence of the specific protease inhibitor. Means± SE (n = 3) and *P<0.05 and **P<0.01. Asterisks in the graphs denote significant differences between the presence and absence of the inhibitors. Bars without asterisk show no significant differences between treatments.

**Fig 6:**
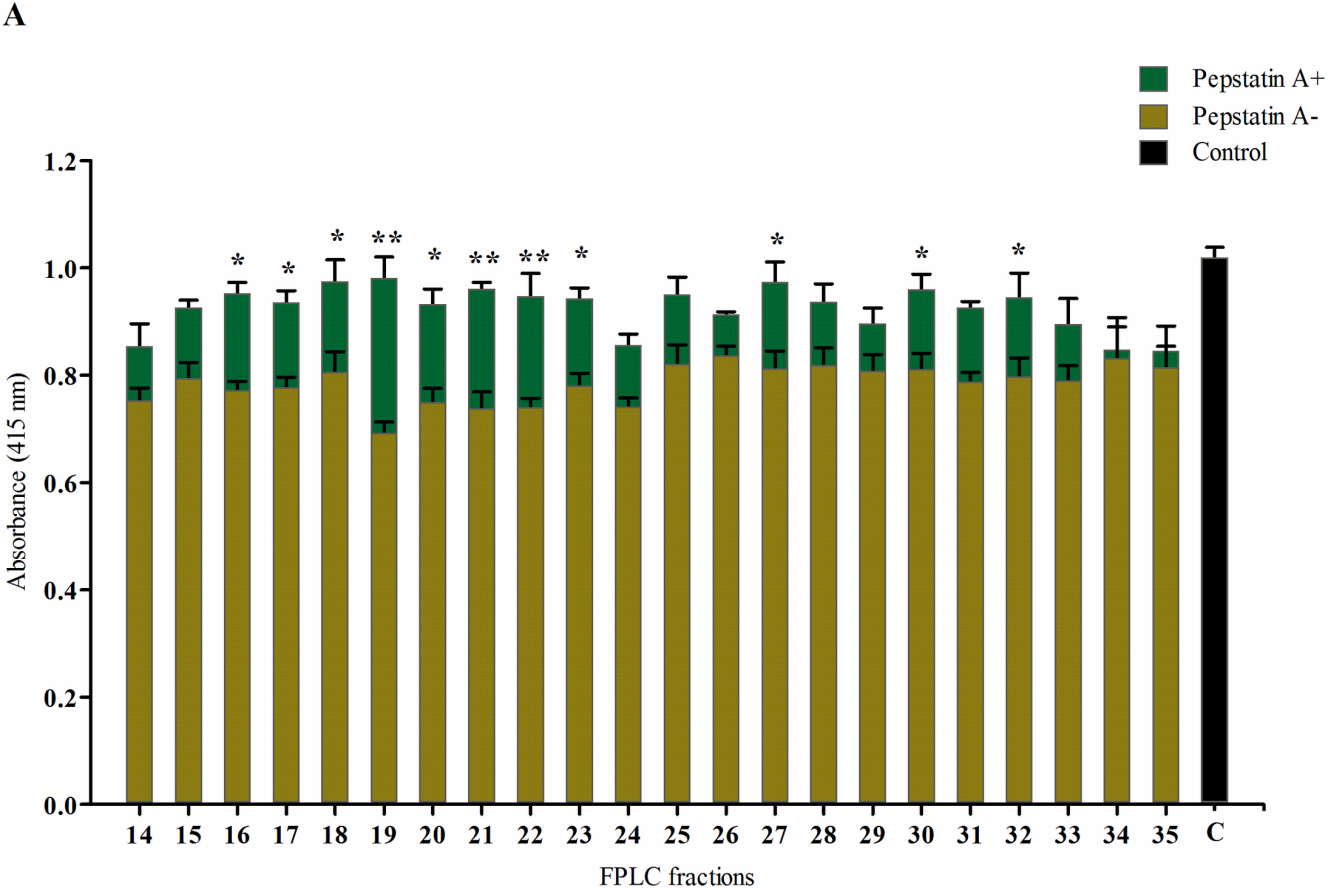
The effect of pepstatin A on the inhibitory activity of the FPLC fractions after pre-incubation at room temperature for 1 hr (P+ with pepstatin; P-, without pepstatin; C, blank sample). Means± SE (n = 3) and **P<0.01 and ***P<0.001. Asterisks in the graph indicate significant differences between the presence and absence of pepstatin A.

In order to test for protease activity of the phytase inhibitor, the gel filtration fractions (14–35, Fig 3) were treated with inhibitors of the main proteases. The effect of the protease inhibitors were first investigated without pre-incubating phytase inhibitor fractions and the specific protease inhibitor. Inclusion of the aspartic proteinase inhibitor pepstatin A significantly reduced the effect of the phytase inhibitor (Fig 5). Without pepstatin A, the phytase activity was inhibited from 17.6 to 31.8% meaning that other potential inhibitors could be contemporary present. When Pepstatin A was added, the reduction was only from 3.5 to 16.8%. The remaining protease inhibitors had no significant effect on the effect of the phytase inhibitor. Pre-incubation of the phytase inhibiting fractions with pepstatin A for 1 h before assaying reduced the effect of the phytase inhibitor even further (Fig 6). Here, phytase activity was increased from 26.7 to 58.3%. Overall, from the results of this section of the study it can be concluded that a significant fraction of the barley grain protein inhibition of *Aspergillus* phytase activity can be attributed to protease activity belonging to the class of aspartic proteinase.

## Discussion

Microbial enzymes including proteases, xylanases and phytases are used as feed additives to increase feed use efficiency and micronutrient bioavailability. In order to increase the efficiency of these enzymes, the effect of enzyme inhibitory components in the feed grains should be taken into account. Proteinaceous and non-proteinaceous inhibitors of microbial proteases and xylanases have been identified and described from different sources including cereal grains [13,20]. The inhibitors have co-evolved with microbial enzymes and contribute to the basal defense response against pathogenic microorganisms. The available information on the identity and levels of the inhibitors has delivered the basis for designing proper scheme in livestock nutrition. In addition, the data has been used for developing resistant cultivars against phytopathogenic organisms [15,21]. Phytate is the major storage compound of phosphate in cereals grains. About 90% of grain phytate is located in the aleurone layer of the grain and constitutes a key target for *A*. *niger* for mobilizing phosphate for growth. In the current study, we for the first time describe how cereal grain components can inhibit *A*. *ficuum* phytase activity.

Previous reports indicated that phytate, metal ions and polyphenols cause the inhibition of plant and microbial phytases. Depending on the type of phytase, the natural substrate phytate can be an inhibitor of the phytase above a certain level. A minimum phytate concentration of 300 μM and 20 mM were described to inhibit maize root [22] and the soybean [23] phytases, respectively. Phytases may require different levels of metal ions for their optimal activity. However, the type and concentration of metal ions in the reaction mixture can affect the activity of phytases. For instance, 5 mM Cu^2+^and Zn^2+^ strongly inhibited the *Schwanniomyces castellii* phytase whereas 5 mM Ca^2+^, Mg^2+^, Mn^2+^and Fe^2+^ slightly inhibited the enzyme activity[24]. Phytases from *A*. *ficuum* [17], *E*. *coli* [25], *Klebsiella terrigena* [26], *Selenomonas ruminantium* [27] were strongly inhibited by 5 mM Cu^2+^, Hg^2+^, Zn^2+^, Fe^2+^ and Fe^3+^. In addition, the effect of metal ions was described for the plant phytases TaPhyIIa2 and HvPhyIIb [28]. The wheat TaPhyIIa2 and barley HvPhyIIb were strongly inhibited by Cu^2+^and Zn^2+^ ions. The inhibition of plant phytases by the polyphenols is described as a physiological phenomenon during germination. The polyphenol phloroglucinol (1, 3, 5-benzenetriol) non-competitively inhibited *Cucurbita maxima* phytase *in vitro* [29].

The crude extracts of all the examined cereals significantly reduced the activity of *A*. *ficuum* phytase. In all cases the phytase activity was inhibited in a dose-dependent manner (Fig 1). The magnitude of the inhibitory effect varies among species and different cultivars of barley and wheat. A similar trend has been reported for xylanases, where the effect of commercial xylanases varies significantly among species and cultivars [13,30]. In the present study, the highest inhibition rates were observed using GPE from maize and *Fusarium* infected wheat. In maize, *Aspergilli* species are the main postharvest pathogens [6]. They cause seedling blight and kernel rot diseases. Previously it has been reported that maize seed proteins inhibit the growth and aflatoxin biosynthesis of *A*. *flavus* [31]. Therefore, maize seeds may have evolved inhibitory compounds against hydrolases of *Aspergilli* fungi, including for phytases.

Induced resistance towards plant pathogens is a known phenomenon [32,33], and broad spectrum induced resistance, where a plant attacked by one pathogen gets resistant towards another pathogen, is also known [34]. Specifically for *Fusarium* it has been demonstrated that infection of tomato with isolates of *Fusarium oxysporum* F. sp. *Cucumerinum* can induce resistance to late blight [35]. In the current study, infection of wheat with Fusarium induced a specific inhibition of phytase activity in a different pathogen (*A*. *ficuum*). Future studies will have to uncover to which level the varying levels of phytase inhibitors in the other tested wheat and barley samples are induced by different biotic stress exposures during cultivation or by genetics not affected by biotic stress.

Michaelis-Menten kinetics and Lineweaver-Burk plots did not reveal a clear mechanism of the inhibition mechanism (**Fig 2**) and in order to test for protease activity of the phytase inhibitor, the protease inhibitors E-64, EDTA, PMSF and pepstatin A were incubated together with phytase and the phytase inhibiting fractions. A strong protease involvement in the phytase inhibition was demonstrated by the reduced inhibition in samples with the aspartic proteinase inhibitor pepstatin A. A 1 h pre-incubation of GPE with pepstatin A significantly enhanced the relative effect of pepstatin A (Fig 6). Based on these results it can be concluded that the significant inhibition of *A*. *ficuum* phytase observed in barley extracts can be attributed protease activity, specifically from an aspartic proteinase.

The presence of inhibitors of *A*. *ficuum* phytase in cereals like wheat, maize, rice and barley has major implications for a range of phytase applications. For feed and food, inhibitors levels may compromise expected bio-availability levels of phosphate and micro-nutrients. This may lead to unexpectedly low or high effects of adding *A*. *ficuum* phytase to food or feed mixtures of not only different cereal species but also between cultivars and within cultivars with different health status. A lower effect in feed will lead to increased secretion of undigested phytate P and minerals into the environment. In case of a higher effect than expected, the addition of feed phosphate and minerals could be reduced. In contrary, the presence of inhibitors of key enzyme activities like phytase in a serious pathogen opens up for new potentials in resistance breeding. Finally, the current study focuses on the inhibition of *A*. *ficuum* phytase. Phytases from other organisms may also be inhibited by cereal proteins. Future studies will have to uncover the overall effect of cereal phytase inhibitors in a food and feed context as well as the potentials in resistance breeding.
